# Reference values of vessel diameters, stenosis prevalence, and arterial variations of the lower limb arteries in a male population sample using contrast-enhanced MR angiography

**DOI:** 10.1371/journal.pone.0197559

**Published:** 2018-06-20

**Authors:** Roberto Lorbeer, Andreas Grotz, Marcus Dörr, Henry Völzke, Wolfgang Lieb, Jens-Peter Kühn, Birger Mensel

**Affiliations:** 1 Department of Radiology, Ludwig Maximilian University Hospital, Munich, Germany; 2 Institute for Community Medicine, University Medicine Greifswald, Greifswald, Germany; 3 Institute of Diagnostic Radiology and Neuroradiology, University Medicine Greifswald, Greifswald, Germany; 4 Department of Internal Medicine B, University Medicine Greifswald, Greifswald, Germany; 5 DZHK (German Centre for Cardiovascular Research), partner site Greifswald, Greifswald, Germany; 6 DZD (German Centre for Diabetes Research), Greifswald, Germany; 7 Institute of Epidemiology, Christian Albrecht University, Kiel, Germany; "INSERM", FRANCE

## Abstract

**Introduction:**

Morphological characterization of leg arteries is of significant importance to detect vascular remodeling triggered by atherosclerotic changes. We determined reference values of vessel diameters and assessed prevalence of stenosis and arterial variations of the lower limb arteries in a healthy male population sample.

**Methods:**

Gadolinium-enhanced magnetic resonance angiography at 1.5 Tesla was performed in 756 male participants (median age = 52 years, range = 21–82 years) of the population-based Study of Health in Pomerania. Vessel diameters were measured in 9 predefined segments of the pelvic and leg arteries and 95th percentiles were used for upper reference values of means of left and right side arteries.

**Results:**

Reference values of vascular diameters decreased from proximal to distal arteries: common iliac = 1.18cm; internal iliac = 0.75cm; external iliac = 1.03cm; proximal femoral = 1.02cm; distal femoral = 0.77cm; popliteal = 0.69cm; anterior tibial = 0.42cm; posterior tibial = 0.38cm; fibular = 0.40cm. Body-surface area indexed reference values increased with age in all segments. A number of 53 subjects (7.0%) had at least one stenosis, mainly in the lower leg arteries anterior tibial (n = 28, 3.7%), posterior tibial (n = 18, 2.4%) and fibular (n = 20, 2.6%). The risk of stenosis increased considerably with age (odds ratio = 1.08; *p*<0.001). The most common arterial variant was type I-A in both legs (n = 620, 82%).

**Conclusion:**

We present reference values for different pelvic and leg artery segment diameters in men that decrease from proximal to distal and increase with age. Stenoses were most prevalent in lower leg arteries and type I-A was the most common variant in the lower leg.

## Introduction

Asymptomatic and symptomatic peripheral arterial diseases (PAD) represent common manifestations of the atherosclerotic disease process and are associated with an increased risk for other manifestations of cardiovascular disease and mortality [1]. It is well established that an adverse cardiovascular risk profile predisposes to PAD. PAD is common in smokers [2], in patients with diabetes mellitus [3] and in obese individuals [4] and predicts overall mortality in the general population, independent of established risk factors [5,6].

To detect vascular remodeling triggered by atherosclerotic changes in the leg arteries at the subclinical status of PAD, the morphological characterization of leg arteries is of significant importance [7]. Imaging diagnostic methods for the evaluation of leg arteries and PAD include digital subtraction angiography, duplex sonography and computed tomography angiography. So far several studies have only investigated single segments of leg arteries by these different methods in patients but not in populations-based samples of healthy subjects [8–12].

Magnetic resonance angiography (MRA) is an alternative method to evaluate leg artery morphology non-radiation-based and is more sensitive and specific to detect stenosis compared to duplex sonography and computed tomography angiography [13]. Until now only few studies have used MRA for investigations of leg arteries in patients [14,15], but not in the general population to establish important reference values for arterial morphology, to assess the subclinical status of PAD and obtain information on artery variants as vascular surgery conditions in the general community.

Therefore, we used MRA examinations of the pelvic and peripheral arteries on both legs to assess reference values for vessel diameters in 9 segments on both sides, the prevalence of stenosis and the prevalence of popliteal artery variants in a large male sample of the population-based Study of Health in Pomerania (SHIP).

## Materials and methods

### Study sample

SHIP consists of two population-based cohorts recruited in the northeast region of Germany. The first cohort (SHIP-0) with 4308 participants aged 20–79 years was surveyed between 1997 and 2001 [16]. The second follow-up after 11 years of the first cohort (SHIP-2) included 2333 participants (1098 men). The second independent cohort (SHIP-TREND) was established in parallel between 2008 and 2012, including 4420 participants (2145 men; age range, 20–79 years) [17]. The two cohorts SHIP and SHIP-TREND were selected from essentially the same area of West Pomerania. Only individuals with German citizenship and main residency in the study area were included. Subjects were sampled equally for each gender and in each of 12 five-year age strata supported by the residents’ registration offices. Subjects were selected in two waves to minimize drop-outs by migration. Selected persons received a maximum of three written invitations with detailed information about the examination. The letters were followed by a telephone call in the case of non-response or by home visits if non-responders could not be contacted by phone.

**Fig 1:**
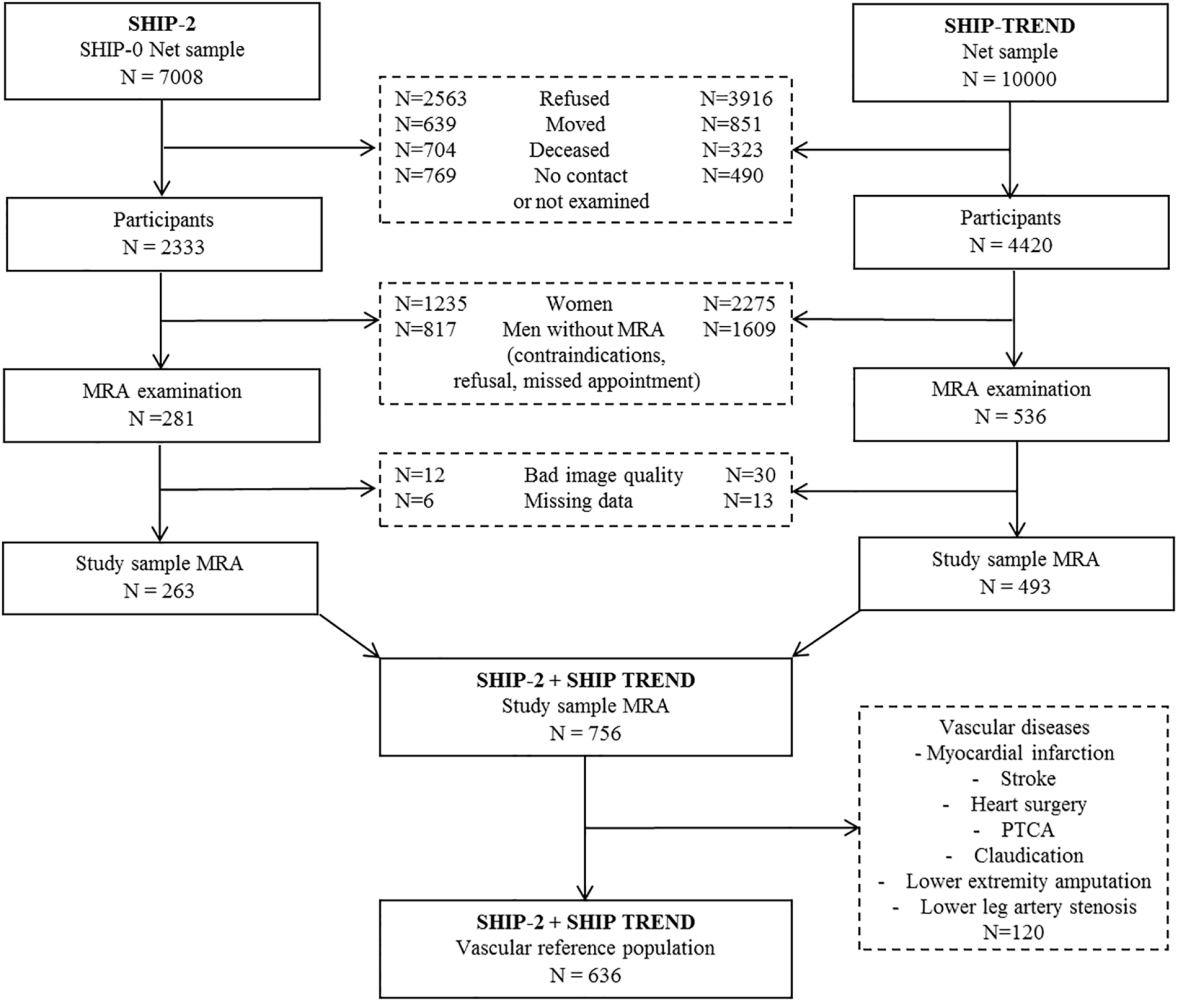
Study sample flow-chart. The development of the whole MRA study sample and the vascular reference population for determination of reference vessel diameters.

Of these two cohorts (SHIP-2 and SHIP-TREND), a total of 817 male subjects were examined by contrast-enhanced MR angiography between 2008 and 2012. Exclusion criteria for the present analyses were insufficient imaging quality (N = 42) and missing data for vessel diameters of the different segments (N = 19). The overall study sample comprised 756 men, aged 21–82 years. To assess reference vessel diameters, further symptomatic subjects with vascular disease including history of myocardial infarction, stroke, heart surgery, percutaneous transluminal coronary angioplasty, claudication, lower extremity amputation or lower leg arteries stenosis (N = 120) were excluded yielding a male asymptomatic reference population of N = 636 (Fig 1).

The study was approved by the Ethics Committee of the University of Greifswald and complies with the Declaration of Helsinki. Written informed consent was provided by all participants.

### MRI examination and vessel measurements

**Fig 2:**
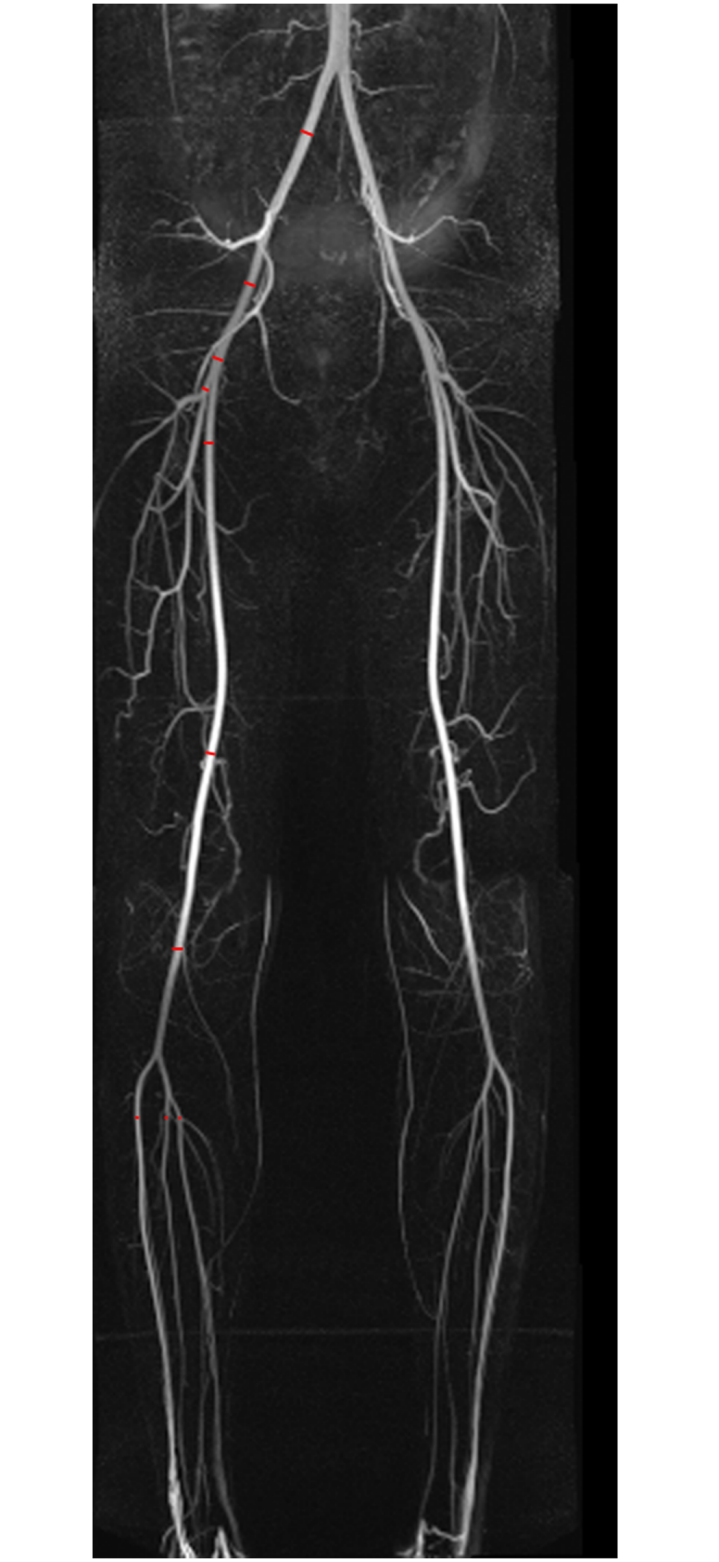
Example of male pelvic and leg arteries. Derived from contrast-enhanced MR angiography in the Study of Health in Pomerania. Red lines correspond to vessel measurements.

MRI was performed on a 1.5 T scanner (Magnetom Avanto; Siemens Healthcare, Erlangen, Germany) with the participant in supine position using phased array surface coils. MRA was done after a previous whole-body MRI [18]. Before contrast medium was applied, renal function had been assessed by estimating the glomerular filtration rate (GFR) using the Modification of Diet in Renal Diseases formula [19]. Participants with an estimated GFR < 60 ml/min/1.73m^2^ and known contrast allergy were excluded. Gadobutrol (Gadovist; Bayer Schering Healthcare, Leverkusen, Germany) with the injection of 0.1 ml per kilogram bodyweight at a flow rate of 0.7 ml/sec followed by 20 ml saline at a rate of 1.2 ml was given via the right or left cubital vein. Two slabs (TR: 248 msec, TE: 90 msec, flip angle: 25°, matrix: 335x512) with a spatial resolution of 2.0x1.0x1.5 mm^3^ for pelvic and femoral arteries and one slab (TR: 255 msec, TE: 90 msec, flip angle: 25°) with a voxel size of 1.4x1.0x1.5 mm were acquired using care bolus technique. The vessel measurements were done at 9 predefined artery segments at each side: 1) common iliac, 2) internal iliac, 3) external iliac, 4) femoral (proximal), 5) femoral (distal), 6) popliteal, 7) anterior tibial, 8) posterior tibial, and 9) fibular (Fig 2).

Imaging analysis was obtained with the Picture Archiving and Communication System (PACS) „IMPAX”(IMPAX 6.5.2.114, Agfa Health Care N.V. 2011, Mortsel, Belgium).

Vessel diameter measurements were taken in T1-weighted contrast enhanced flash sequences in the coronary layer. For overview purposes maximum intensity projection images were used additionally. For each predefined segment the image layer with the maximum vessel diameter was explored and the inner diameter perpendicular to the vessel wall was measured.

All images used for the present analysis were evaluated visually for stenosis in the pelvic and leg arteries by one trained observer. Stenoses were graded compared to the assumed normal vascular diameter in the particular segment. The degree of stenosis (narrowing of the diameter) in each of the segments was categorized as grade 1 (30–49%), grade 2, (50–99%) and grade 3 (100%, thrombosis).

Popliteal arterial variants were classified according to the system suggested by Kim et al. [20]. The classification system includes 10 categories (type I-A)-(type III-C) characterizing the branching of the popliteal artery and the arterial supply to the foot with type I-A as the normal type where the anterior tibial artery is the first arterial branch followed by the tibioperoneal artery that bifurcates into the peroneal and posterior tibial arteries [20].

### Statistical analyses

Characteristics of the study population and non-participants are displayed as median (25^th^ and 75^th^ percentile) and numbers (percent values) if applicable. Differences between study population (with available data on MRA) and non-participants were tested using either χ2-test (nominal data) or Mann-Whitney U-test (interval data). For further analyses, inverse observation probability weights were calculated by inverse participation probabilities predicted by logistic regression including the explanatory variables age, smoking status, body mass index, hypertension and diabetes that have been defined elsewhere [21]. Comparisons of left and right vessel diameters were done by testing differences of mean values (standard deviation) by paired t-tests.

The mean of the left and right vessel diameter was calculated to assess upper reference values using 95^th^ percentiles of the different artery segments for three age groups (20–39 years, 40–59 years, 60–89 years). Furthermore, continuous age-related reference values were estimated for body surface area (BSA)-indexed vessel diameters by providing intercepts and β-coefficients using weighted quantile regression for the 95^th^ percentiles. BSA was calculated according to the Du Bois formula [22]: (BSA = 0.007184×(height in cm)^0.725^×(weight in kg)^0.425^. These latter analyses and the prevalence analyses of stenosis and arterial variants were performed separately for the right and the left leg. The prevalence of stenosis and arterial variants were estimated after weighting the study population by characteristics mentioned above. McNemar’s χ2-test was used to compare stenosis rate between left and right legs and the association of age and other risk factors with risk of stenosis was assessed by logistic regression providing odds ratios. A value of p<0.05 was considered statistically significant. Statistical analyses were performed using Stata 13.1 (Stata Corporation, College Station, TX, U.S.A.).

## Results

The study sample of 756 male participants had a median age of 52 years, a proportion of current smokers of 19%, a median body mass index of 27.7 kg/m^2^ and a proportion of 53% were hypertensive (Table 1). In comparison to non-participants, study participants were younger, had a higher proportion of never-smoker and were less often hypertensive.

**Table 1:** Selected characteristics of the male study population and male non-participants.

Characteristics	Study population n = 756	Non-participants n = 2487	p-value*
Age in years	52 (43; 64)	56 (43; 68)	<0.001
Smoking status			<0.001
Never-smoker	244 (32.4%)	584 (23.6%)	
Ex-smoker	367 (48.7%)	1174 (47.5%)	
Current smoker	143 (19.0%)	714 (28.9%)	
Body mass index, kg/m^2^	27.7 (25.5; 30.3)	28.4 (25.7; 31.5)	<0.001
Body surface area (m^2^)	2.03 (1.93; 2.15)	2.04 (1.92; 2.16)	0.649
Systolic blood pressure, mmHg	134 (124; 144)	135 (125; 147)	0.050
Diastolic blood pressure, mmHg	81 (75; 87)	80 (73; 87)	0.006
Hypertension	400 (53.1%)	1498 (60.4%)	<0.001
Diabetes	65 (8.7%)	424 (17.4%)	<0.001
Statins	83 (11.0%)	480 (19.3%)	<0.001
Antithrombotic Agents	84 (11.1%)	577 (23.2%)	<0.001
ACE inhibitors	93 (12.3%)	518 (20.8%)	<0.001

Data are given as numbers (percentage) or as median (25^th^ and 75^th^ percentile)

* χ^2^-test (nominal data) or Mann-Whitney U-test (interval data)

### Reference values for vessel diameters

In the reference population (N = 636), upper reference values (95^th^ percentiles) of vascular diameters decreased from proximal to distal arteries: common iliac artery, 1.18cm (interquartile range: 0.89–1.04); internal iliac artery, 0.75cm (0.45–0.59); external iliac artery, 1.03cm (0.72–0.88); proximal femoral artery, 1.02cm (0.73–0.88); distal femoral artery, 0.77cm (0.52; 0.64); popliteal artery, 0.69cm (0.49; 0.59); anterior tibial artery, 0.42cm (0.31; 0.35); posterior tibial artery, 0.38cm (0.30; 0.32); fibular artery, 0.40cm (0.29; 0.32) (Table 2).

**Table 2:** Median vessel diameters and upper reference values (95th percentile) of different artery diameters according to age groups: 21–39 years (N = 137), 40–59 years (N = 334) and 60–81 years (N = 165).

Artery	Age (years)	Artery diameter (mean of left and right vessel diameter) cm	
		Median (25^th^, 75^th^)	Reference value (95^th^ percentile)
common iliac		0.97 (0.89; 1.04)	1.18
	20–39	0.93 (0.87; 1.00)	1.09
	40–59	0.97 (0.88; 1.05)	1.18
	60–81	1.01 (0.93; 1.09)	1.22
internal iliac		0.51 (0.45; 0.59)	0.75
	20–39	0.50 (0.44; 0.55)	0.62
	40–59	0.51 (0.45; 0.58)	0.70
	60–81	0.56 (0.50; 0.67)	0.84
external iliac		0.81 (0.72; 0.88)	1.03
	20–39	0.79 (0.71; 0.84)	0.98
	40–59	0.80 (0.71; 0.88)	1.02
	60–81	0.83 (0.76; 0.92)	1.07
femoral (prox.)		0.81 (0.73; 0.88)	1.02
	21–39	0.77 (0.71; 0.83)	0.97
	40–59	0.80 (0.72; 0.87)	1.01
	60–81	0.84 (0.78; 0.91)	1.03
femoral (dist.)		0.59 (0.52; 0.64)	0.77
	21–39	0.59 (0.50; 0.61)	0.74
	40–59	0.59 (0.52; 0.64)	0.73
	60–81	0.59 (0.59; 0.69)	0.79
popliteal		0.52 (0.49; 0.59)	0.69
	21–39	0.50 (0.45; 0.55)	0.65
	40–59	0.52 (0.49; 0.59)	0.68
	60–81	0.59 (0.50; 0.64)	0.79
anterior tibial		0.35 (0.31; 0.35)	0.42
	21–39	0.35 (0.32; 0.35)	0.43
	40–59	0.35 (0.31; 0.35)	0.41
	60–81	0.35 (0.31; 0.37)	0.43
posterior tibial		0.31 (0.30; 0.32)	0.38
	21–39	0.31 (0.29; 0.31)	0.38
	40–59	0.30 (0.30; 0.31)	0.38
	60–81	0.31 (0.30; 0.33)	0.39
fibular		0.30 (0.29; 0.32)	0.40
	21–39	0.30 (0.29; 0.33)	0.40
	40–59	0.30 (0.29; 0.31)	0.39
	60–81	0.30 (0.29; 0.33)	0.40

BSA-indexed reference values increased with age in all segments, specifically in the upper leg arteries (Table 3). E.g., older age was associated with larger left reference diameter of common iliac artery (β = 0.00229, p<0.001), internal iliac artery (β = 0.00290, p<0.001) and external iliac artery (β = 0.00248, p<0.001).

**Table 3:** Parameters for calculation of reference values (95^th^ percentile) for body surface area-indexed artery diameters according to age.

	BSA indexed artery diameter (95^th^ percentile) cm/m^2^		
	Intercept	β(age)	p-value*
Artery (left)			
common iliac	0.44444	0.00229	<0.001
internal iliac	0.21815	0.00290	<0.001
external iliac	0.37285	0.00248	<0.001
femoral (prox.)	0.40957	0.00167	0.017
femoral (dist.)	0.28461	0.00160	<0.001
popliteal	0.26946	0.00159	<0.001
anterior tibial	0.18772	0.00043	0.039
posterior tibial	0.14702	0.00077	<0.001
fibular	0.17649	0.00031	0.277
Artery (right)			
common iliac	0.46045	0.00228	<0.001
internal iliac	0.21419	0.00249	<0.001
external iliac	0.40033	0.00180	0.001
femoral (prox.)	0.39878	0.00172	0.003
femoral (dist.)	0.27089	0.00181	<0.001
popliteal	0.25155	0.00170	0.003
anterior tibial	0.18823	0.00039	0.175
posterior tibial	0.15903	0.00055	0.010
fibular	0.17224	0.00044	0.093

*Parameters are from weighted quantile regression; BSA, body surface area

The upper reference value for the common iliac artery diameter of a 52-year-old man with a BSA of 2.03m^2^ (representing median values of the study sample) was exemplarily 1.14 cm ((0.44444+0.00229*52)*2.03).

For the following segments, left vessel diameters were larger than right vessel diameters: internal iliac artery (left = 0.54 cm; right = 0.53 cm, p = 0.004); external iliac artery (0.81; 0.80, p<0.001); proximal femoral artery (0.81; 0.80, p<0.001); popliteal artery (0.54; 0.53, p<0.001) and anterior tibial artery (0.34; 0.34, p<0.001) (Table 4). No differences in vessel diameters between left and right side could be observed for the other segments.

**Table 4:** Comparisons of left and right artery diameters.

Artery	Left diameter, cm	Right diameter, cm	p-value*
common iliac	0.970 (0.129)	0.970 (0.130)	0.802
internal iliac	0.538 (0.123)	0.529 (0.108)	0.004
external iliac	0.809 (0.132)	0.797 (0.130)	<0.001
femoral (prox.)	0.812 (0.120)	0.803 (0.117)	<0.001
femoral (dist.)	0.593 (0.093)	0.594 (0.091)	0.601
popliteal	0.543 (0.091)	0.534 (0.095)	<0.001
anterior tibial	0.343 (0.046)	0.336 (0.046)	<0.001
posterior tibial	0.309 (0.038)	0.309 (0.038)	0.992
fibular	0.308 (0.039)	0.310 (0.045)	0.126

Data are given as mean (standard deviation)

* paired t-test

### Prevalence of stenosis

A number of 53 subjects (7.0% of 756) had at least one stenosis (1: n = 19, 2: n = 19, 3: n = 9, 4: n = 4, 5: n = 1, 6: n = 1), mainly in the lower leg arteries anterior tibial (n = 28, 3.7%), posterior tibial (n = 18, 2.4%) and fibular (n = 20, 2.6%) (Table 5, S1 Table). Of all stenosis, 24% were of low severity (grade 1), 34% were grade 2 and 42% were severe (grade 3). The stenosis prevalence did not differ significantly between left and right vessel of each artery. The prevalence of stenosis increased considerably with age (OR = 1.07; p<0.001), diabetes (OR = 3.58; p = 0.001) and antithrombotic medication (OR = 2.59; p = 0.015) (S2 Table).

**Table 5:** Stenosis prevalence in different artery segments.

Artery	Stenosis	
	Left leg	Right leg
common iliac	0	1 (0.1%)
internal iliac	1 (0.2%)	0
external iliac	1 (0.4%)	0
femoral (prox.)	1 (0.2%)	0
femoral (dist.)	4 (0.8%)	3 (0.7%)
popliteal	0	1 (0.0%)
anterior tibial	23 (3.1%)	24 (3.6%)
posterior tibial	18 (3.0%)	12 (2.0%)
fibular	12 (1.8%)	15 (2.1%)

Data are numbers and weighted proportions (N = 750)

### Popliteal arterial variants

Most of the participants had arterial variant type I-A in both legs (n = 620, 82%) followed by right type I-A/left type I-B (n = 18, 2.4%) and right type I-A/left type II-A1 (n = 17, 2.2%) (Table 6). In 85% of the participants the arterial variants in the left and right leg were the same, whereas in 15% of the participants they differed between left and right leg. The arterial variants type II-C and type III-B were not observed in our sample.

**Table 6:** Distribution of arterial variants in the left and right leg.

Arterial Variant	Left leg	Right leg
Type I-A	664 (88.2%)	682 (90.1%)
Type I-B	23 (2.9%)	19 (2.5%)
Type I-C	5 (0.6%)	5 (0.5%)
Type II-A1	23 (3.0%)	17 (2.2%)
Type II-A2	9 (1.2%)	5 (0.7%)
Type II-B	16 (2.0%)	12 (1.4%)
Type II-C	0	0
Type III-A	15 (2.1%)	15 (2.0%)
Type III-B	0	0
Type III-C	1 (0.0%)	1 (0.0%)

Data are numbers and weighted proportions

Type I-A: the usual pattern of popliteal arterial branching and arterial supply to the foot.

Type I-B: trifurcation: the anterior, peroneal, and posterior tibial arteries arise at the same point without an intervening tibioperoneal trunk.

Type I-C: the posterior tibial artery is the first branch. The anterior tibial and peroneal arteries arise from a common trunk.

Type II-A-1: the anterior tibial artery arises above the knee joint and has a straight course in its proximal segment.

Type II-A-2: the anterior tibial artery arises above the knee joint but takes a medial swing, presumably resulting from its passage anterior to the popliteus muscle.

Type II-B: the posterior tibial artery arises at the level of the knee joint.

Type II-C: the peroneal artery arises above the knee joint.

Type III-A: the posterior tibial artery is hypoplastic and the peroneal artery is large. At the ankle, the distal posterior tibial artery is replaced

to the peroneal artery.

Type III-B: the anterior tibial artery is hypoplastic and the peroneal artery is large. At the ankle, the dorsalis pedis artery is replaced to the peroneal artery.

Type III-C: both the anterior tibial artery and the posterior tibial artery are hypoplastic. At the ankle the dorsalis pedis and posterior tibial arteries are replaced to the peroneal artery.

## Discussion

We report key parameters of vascular morphology (upper reference vessel diameters, prevalence of stenosis and popliteal artery variants) of pelvic and leg arteries in the general male population, based on MRA examinations. Specifically, we provide these parameters for 9 vascular segments on each side. As expected, reference artery diameters decreased from proximal to distal segments and increased with age. For most vessel segments, left artery diameter was significantly larger than right diameter.

In this male sample from the general population, stenoses in the pelvic and leg arteries were relatively rare (overall: 7%), but slightly more prevalent in the lower leg arteries. The prevalence of arterial variants was represented with type I-A being most common with around 90% in each leg.

So far, artery diameters have been investigated by imaging methods only for few segments in smaller clinical samples. The study of Horejs et al. calculated mean aortic diameters in ten years age groups of 130 male patients without vascular complaints and revealed diameters of 1.17 cm, 1.17 cm; 1.05 cm, 1.04 cm for the common iliac arteries (right and left) and the common femoral arteries (right and left), respectively in the age group of 50–60 years using computed tomography [9]. In another study, the common femoral artery of a chosen site was characterized in 59 healthy male subjects aged 8 to 81 years by mean and median diameters of 9.8 mm and 9.7 mm using an ultrasound examination [12]. In the same sample of the latter study, mean diameter of the popliteal artery revealed 8.4 mm in the oldest male age group (mean age: 66.8 years) [11]. The reported mean values of artery diameters are larger compared to our study and are very close to or even higher than our estimated upper reference values. Reference values were not reported in the smaller studies. Our reference population is from the male healthy general population without clinical evidence of vascular disease and provides normal upper reference values of vessel diameters that are expected to be different from values from patients or volunteers from the hospital. Further possible reasons for different vessel diameter levels between studies might arise from different imaging methods, measurements methods (inner diameter vs. outer diameter) or the use of different age groups.

The decrease of arterial diameters from proximal to distal segments, observed in our analyses, is consistent with prior studies [10–12,23]. These studies reported larger diameters for the anterior tibial artery of the normal arterial variant compared to the posterior tibial artery [23] and larger diameters for the common femoral artery compared to the popliteal diameter [11,12].

An increasing artery diameter with age is well established in the literature [10–12]. In our study, all 9 analysed artery segments showed positive associations between BSA-indexed artery diameter reference values and age. A dilation of the upper leg arteries with older age was also demonstrated for the femoral and the popliteal artery by Sandgren et al. [11,12] and Macci et al. [10]. The latter study could also demonstrate a positive association between the diameter of the posterior tibial artery and age. Strong evidence is also available for the dilation of thoracic and abdominal aortic diameters with increasing age [24,25].

Diameter differences between left and right leg arose in 5 of 9 segments in the present study. Mean left diameters of the arteries internal iliac artery, external iliac, femoral (proximal), popliteal and anterior tibial were consistently larger compared to mean right diameters of these arteries. This result is not supported by the literature. In a sample of 152 specimens of lower limbs from amputated legs of 124 male and female patients with critical limb ischaemia, right artery diameters were observed to be larger compared to left diameters, especially for the posterior tibial artery [23]. The latter results were in line with the results of a study with 100 healthy male and female subjects with no history of occlusive arterial disease of the lower extremities, that measured the principal arteries of the lower extremities by Doppler ultrasound and revealed non-significant larger right arteries with an exception of significance for the common femoral arteries and the tibial posterior arteries [10]. Other studies have not differentiated between left and right artery diameters [11,12]. Differences between left and right leg artery diameters could be relevant in association with leg dominance. It can be assumed that in most participants the right leg is the dominant one [26] resulting in more muscle mass which can lead to a compression of the vessels to a certain degree. However, little is known about the prevalence of the dominant leg in the general population, and in our study such data were not available to further explain this finding.

To the best of our knowledge, this is the first study investigating stenosis prevalence in single pelvic and leg arteries by using MRA. In this relatively healthy sample from the general population, the overall stenosis prevalence was 7.0% with the highest prevalence in the lower leg arteries anterior tibial, posterior tibial and fibular. As expected, we observed that stenosis was significantly commonly present in older ages. This finding is in line with studies demonstrating an increasing PAD prevalence measured by ABI of the anterior and posterior tibial arteries for older ages [27].

In 1989, Kim et al. developed an angiographic classification system for popliteal arterial variants using femoral arteriograms from 495 extremities [20]. The normal variant (type I-A: the anterior tibial artery is the first arterial branch followed by the tibioperoneal artery that bifurcates into the peroneal and posterior tibial arteries) could be found in 92.2% of the extremities followed by type I-B (2.0%) and type II-A1 (3.0%). According to the distal branching pattern, type III-A (3.8%) revealed the most prevalent non-normal variant [20]. A similar study from 2006 investigating 1037 lower limbs in 568 patients confirmed the latter results regarding type I-A (90.7%), type I-B (3.2%) and type II-A1 (2.1%) while the prevalence for type II-A2 was slightly higher (2.4%) and for type III-A lower (0.8%) [8]. A comparison between time-resolved and bolus-chase MRA in 53 legs of 27 patients supported the use of MRA for investigating the branching pattern of leg arteries [14].

In the mentioned patient studies, sex distribution of the analyzed extremities is unclear. In our study from the population, we revealed a similar prevalence for the different variants in men using MRA. In addition, we provide prevalence information separated for the left and right leg and detected a significant proportion of subjects with different left and right arterial variants (15%).

For the first time, a wide range of segments of left and right pelvic and leg arteries could be investigated simultaneously by MRA examination in a large male sample. The MRA examination was part of a whole-body MRI examination within two cohorts of a population-based study that has established high quality standards [17]. Therefore, the present findings represent preliminary results for further potential association studies with either correlates of the large basic examination program or other whole-body MRI parameters.

Our study is limited by a low MRA response (23%) relative to the overall study sample (sub-response to the whole body MRI examination was around 50%). The reasons for the response difference might be due to a long examination time (in combination with the previous whole-body scan) or due to the need for applying contrast agents. Although the overall sample size of this study was reasonable for providing reference values of vessel diameters, the power for stenosis prevalence investigation was limited.

The MRA sample was slightly younger with lower proportion of current smokers and with lower body mass index and it was a bit healthier regarding hypertension and diabetes mellitus compared to non-participants. However, we provided inverse observation probability weighted estimates for reference value parameters and prevalence to allow a generalization of our findings based on the initial study sample. By design, the MRA examination was only offered to men. Women were examined instead by MR mammography within a time-consuming whole-body MRI protocol.

We cannot exclude that there was a possible slight over-estimation of the MRA derived vessel diameters as recently reported since our study context did not comprise the validation of vessel diameter measurements, e.g. macroscopic by pathology or visually by gold standard digital subtraction angiography [15,28]. In addition, we cannot exclude that also the degree of stenosis especially on the lower leg vessels was over-estimated by MRA in our study as reported before [29,30]. To what extent selected absolute or indexed diameters are suitable as clinical parameters to characterize chronic vascular wall diseases such as arteriosclerosis should be the object of further follow-up studies.

In conclusion, this is the first study examining pelvic and leg arteries by MRA in a male sample from the general population. We present upper reference values for different pelvic and leg arterial segment diameters in men. The reference vessel diameters decreased from proximal to distal and increased with age. Stenoses were relatively scarce overall, but more prevalent in lower leg arteries as compared to upper leg and pelvic arteries. With respect to popliteal arterial variants, arterial variant type 1-A was most common.

## Supporting information

S1 Table Numbers of stenosis and affected artery segments. Data are numbers.
(DOCX)

S2 Table Multivariable adjusted associations between risk factors and stenosis prevalence of the lower limb arteries. Odds ratios are from multivariable adjusted logistic regression.
(DOCX)
