# Supplementary material for: Reference values of vessel diameters, stenosis prevalence, and arterial variations of the lower limb arteries in a male population sample using contrast-enhanced MR angiography
Source: PLoS One. 2018 Jun 20;13(6):e0197559. doi: 10.1371/journal.pone.0197559 (PMC6010244; doi:10.1371/journal.pone.0197559)
Supplement: S1 Table — Data are numbers. (DOCX) [file pone.0197559.s001.docx]

**S1 Table. Numbers of stenosis and affected artery segments.**

| **Artery** |  | **Number of Stenosis** | | | | | |
| --- | --- | --- | --- | --- | --- | --- | --- |
|  |  | **1** | **2** | **3** | **4** | **5** | **6** |
|  |  | N=19 | N=19 | N=9 | N=4 | N=1 | N=1 |
| common iliac | left | 2 | 0 | 1 | 0 | 0 | 0 |
|  | right | 0 | 0 | 2 | 0 | 0 | 0 |
| internal iliac | left | 0 | 0 | 0 | 0 | 0 | 1 |
|  | right | 0 | 0 | 0 | 0 | 0 | 0 |
| external iliac | left | 0 | 0 | 2 | 0 | 0 | 0 |
|  | right | 0 | 0 | 0 | 0 | 0 | 0 |
| femoral (prox.) | left | 0 | 1 | 0 | 0 | 0 | 0 |
|  | right | 0 | 0 | 0 | 0 | 0 | 0 |
| femoral (dist.) | left | 1 | 1 | 0 | 0 | 0 | 1 |
|  | right | 0 | 2 | 1 | 0 | 0 | 0 |
| popliteal | left | 0 | 0 | 0 | 0 | 0 | 0 |
|  | right | 0 | 0 | 0 | 0 | 0 | 0 |
| anterior tibial | left | 1 | 9 | 5 | 4 | 1 | 1 |
|  | right | 4 | 9 | 5 | 3 | 1 | 1 |
| posterior tibial | left | 1 | 4 | 4 | 3 | 1 | 1 |
|  | right | 2 | 3 | 2 | 4 | 0 | 1 |
| fibular | left | 4 | 4 | 2 | 0 | 1 | 0 |
|  | right | 4 | 5 | 3 | 2 | 1 | 0 |

Data are numbers
