# Supplementary material for: Reference values of vessel diameters, stenosis prevalence, and arterial variations of the lower limb arteries in a male population sample using contrast-enhanced MR angiography
Source: PLoS One. 2018 Jun 20;13(6):e0197559. doi: 10.1371/journal.pone.0197559 (PMC6010244; doi:10.1371/journal.pone.0197559)
Supplement: S2 Table — Odds ratios are from multivariable adjusted logistic regression. (DOCX) [file pone.0197559.s002.docx]

**S2 Table. Multivariable adjusted associations between risk factors and stenosis prevalence of the lower limb arteries.**

| **Risk factors** | **Stenosis** |  |
| --- | --- | --- |
|  | **OR (95%CI)** | **p Value** |
| Age | 1.07 (1.03; 1.10) | <0.001 |
| Smoking status |  |  |
| Never-smoker | 1 |  |
| Ex-smoker | 1.17 (0.56; 2.45) | 0.667 |
| Current smoker | 2.51 (0.98; 6.43) | 0.056 |
| Body mass index | 0.95 (0.86; 1.05) | 0.288 |
| Hypertension | 1.09 (0.52; 2.29) | 0.816 |
| Diabetes | 3.58 (1.63; 7.87) | 0.001 |
| Statins | 0.69 (0.28; 1.74) | 0.432 |
| Antithrombotic Agents | 2.59 (1.20; 5.56) | 0.015 |
| ACE inhibitors | 1.09 (0.48; 2.50) | 0.839 |

Odds ratios are from multivariable adjusted logistic regression
